# A rare case of isolated ACTH deficiency associated with a Rathke's Cleft Cyst and a review of the literature

**DOI:** 10.1002/ccr3.8026

**Published:** 2023-10-09

**Authors:** Hamza Azam, Bernard Tuch

**Affiliations:** ^1^ Liverpool Hospital Liverpool New South Wales Australia; ^2^ Monash University Clayton Victoria Australia

**Keywords:** endocrinology and metabolic disorders, neurology, neurosurgery

## Abstract

A 78‐year‐old man was referred to clinic due to a 5‐year history of weight loss, lethargy, and pathology showing hyponatremia. In the year prior, he had a hospital admission for symptomatic hyponatremia. MRI brain during that admission showed a 1–2 mm pituitary lesion of unknown significance. Testing during this presentation revealed hypocortisolism with ACTH deficiency. Progress MRI brain revealed the presence of a Rathke's Cleft Cyst (RC). Medical management with glucocorticoids resulted in symptomatic and biochemical parameter improvement. To our knowledge this is the first reported case of isolated ACTH deficiency in the setting of a RC.

## Graphical abstract

A Rathke cleft cyst causing isolated ACTH deficiency.

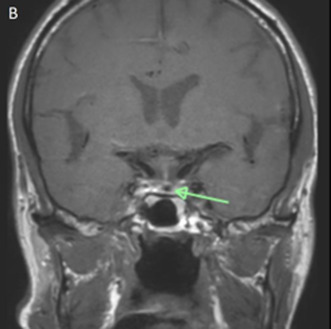

## 1 BACKGROUND

Rathke's Cleft Cyst (RC) is a lesion which derives from the Rathke pouch, a component involved in normal pituitary development. Maldeveloped remnants of this pouch can give rise to cysts, of which the most common are RC.^1^ This is the same structure from which craniopharyngiomas arise. RC can be associated with pituitary dysfunction (including panhypopituitarism) and has been reported to cause apoplexy, oculomotor palsy, and adrenal crises^2^, ^3^, ^4^, ^5^;however, this is the first report to show where RC has been associated with an isolated Adrenocorticotropic hormone (ACTH) deficiency. Given this unusual presentation, our case was initially thought to be of Addison's disease because of the low serum cortisol, then Syndrome of Inappropriate Anti‐Diuretic Hormone Secretion (SIADH) because of hyponatremia until a repeat MRI brain showed typical findings of RC. We looked at the literature to find why only corticotroph cells were affected in our patient.

## 2 CASE PRESENTATION

A 78‐year‐old male, who had been living alone for many years, was referred to clinic due to a 15 kg history of weight loss over the past 5 years, lethargy and hyponatremia. He was noted to be a poor eater. There was no associated dizziness. He did not report any visual disturbances. His past medical history included a diagnosis of essential tremor and he was not taking regular medications.

One year prior he required hospital admission after an elective surveillance colonoscopy, due to nausea, vomiting, and hyponatremia (sodium 119 mmoL/L). In this admission Addison's disease was initially suspected; however, this was ruled out with an early morning cortisol of 210 nmoL/L (normal range: 150–250), a normal short synacthen test and normokalemia. Thyroid function tests and ACTH level were in the normal range. An MRI brain showed a pituitary gland of normal size with a 1–2 mm area of hypointensity on the postero‐inferior aspect of the gland which was thought to be either a microadenoma or artefactual. His presentation was thought to be due to bowel preparation inducing SIADH.

Examination in clinic showed a weight of 58.2 kg and BMI of 20.9. There was asymptomatic hypotension (90/59) with no significant postural drop. Other vital signs were within normal limits. There was no evidence of hyperpigmented skin.

## 3 INVESTIGATIONS

Repeat bloods revealed hyponatremia (132 mmoL/L), hypocalcaemia 2.11 mmoL/L corrected (normal range: 2.15–2.55 mmoL/L), and anemia 120 g/L (normal range 128–175 g/L). Serum potassium and ferritin were within the normal range; however, the serum cortisol was low at 49 nmoL/L. ACTH was inappropriately low at 1.5 pmoL/L (normal: <12.1 pmoL/L), with the rest of the anterior pituitary normal (Table 1) suggesting isolated ACTH deficiency.

**TABLE 1:** Pituitary panel investigations.

Parameter	Level	Reference range
Growth hormone (GH)	0.4 mIU/L	0–15 mIU/L
Insulin‐like Growth Factor 1 (IGF1)	11 nmoL/L	7–28 nmoL/L
Free T4	13.1 pmoL/L	10.0–20.0 pmoL/L
Free T3	4.8 pmoL/L	2.3–5.7 pmoL/L
Thyroid stimulating hormone (TSH)	4.08 mIU/L	0.4–5 mIU/L
Luteinizing hormone (LH)	4.2 IU/L	0.6–1.2 IU/L
Follicle stimulating hormone (FSH)	1.9 IU/L	1.0–1.2 IU/L
Prolactin	452 mIU/L	65–500 mIU/L

**FIGURE 1:**
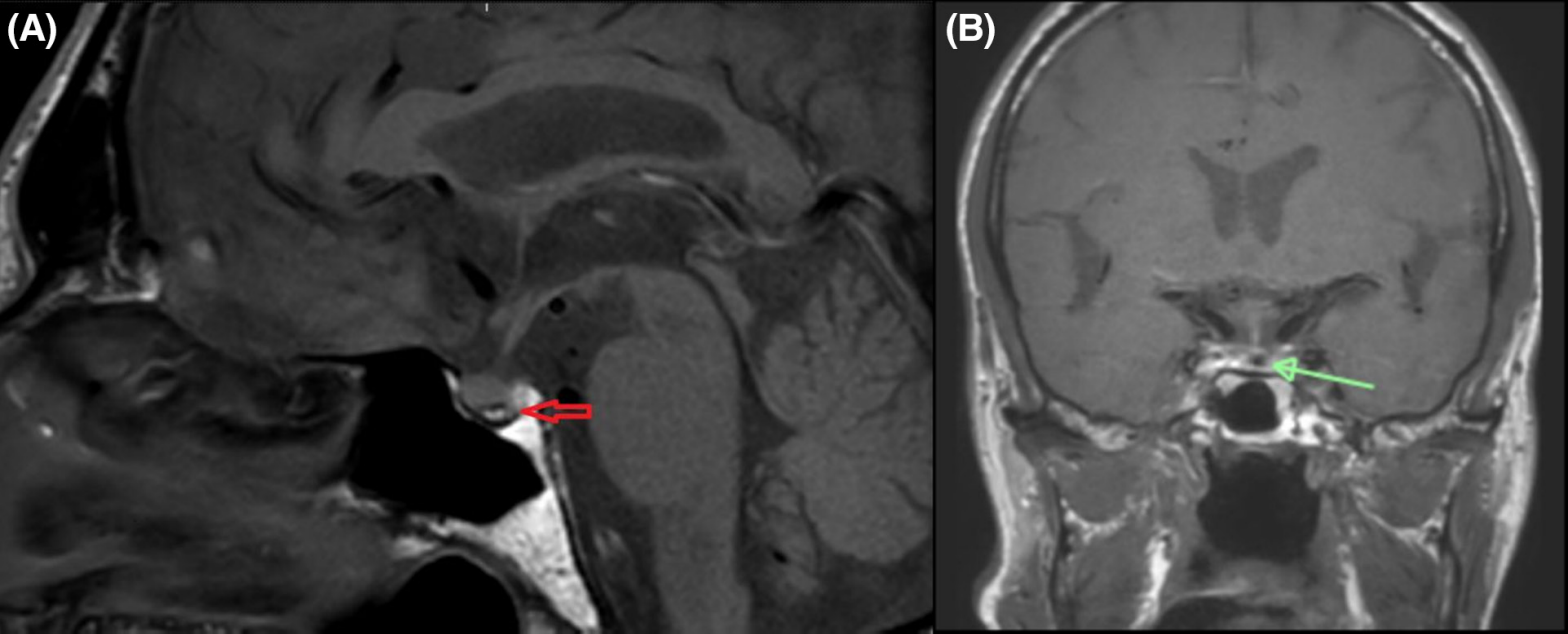
Sagittal (A) and coronal (B) T1 weighted MRI brain showing cystic lesion.

A repeat MRI brain was therefore performed showing a 2.5 × 2.4 × 5.2 mm well‐circumscribed cystic lesion with both low and high‐signal intensities on T1 (Figure 1A,B) and hypointense signal on T2 in keeping with a RC. Features of autoimmune hypophysitis were not seen. On comparison to the MRI brain performed an year prior, the RC had grown in size.

## 4 TREATMENT

Due to isolated ACTH deficiency, with evidence that glucocorticoid replacement therapy reduces RC size,^5^, ^6^ non operative management was pursued with prednisone 5 mg daily. Given improvement to symptoms with prednisone therapy, drainage of RC was avoided to prevent operative complications for a condition responding to pharmacotherapy. Prednisone was later decreased to 2 mg and subsequently 1 mg daily due side effects on mood. To address the weight loss and hypocalcaemia the patient was commenced on nutritional and calcium supplementation.

## 5 OUTCOME AND FOLLOW‐UP

At 5 months follow‐up the patient's weight improved to 62.7 kg with a BMI of 22.5, and at 13 months to 66.1 kg and 23.7, respectively. The patient had tolerated low dose prednisone reasonably well with improvement to his fatigue. Biochemical parameters also showed improvement with morning cortisol at 209 nmoL/L and ACTH at 2.9 pmoL/L. Calcium levels improved to 2.3 mmoL/L, hemoglobin to 133 g/L, and levels of the rest of the pituitary hormones remained normal.

At 6 months follow‐up, the patient ceased taking Prednisone and became weak again, with low serum cortisol 48 nmoL/L, ACTH 1.9 pmoL/L, sodium 131 mmoL/L and hemoglobin 118 g/L. Symptoms improved and biochemical parameters returned to normal when Prednisone 1 mg daily was re‐introduced.

## 6 DISCUSSION

Our case shows a patient with ACTH deficiency in the setting of a RC who responded well to prednisone therapy. He had a relapse of symptoms upon cessation of prednisone. Subsequent recommencement of prednisone led to improving symptoms. To differentiate between secondary and tertiary hypoadrenalism a corticotropin‐releasing hormone stimulation test may have proven useful. However due to logistical difficulties in obtaining the test, it was not performed. Based on the presence of a mass affecting the pituitary gland, it is highly likely the cause of the isolated ACTH deficiency was secondary.

ACTH deficiency presents similarly to glucocorticoid deficiency with symptoms of weight loss, anorexia, nausea, vomiting, postural dizziness and sometimes with symptomatic hypoglycaemia (more commonly seen in secondary adrenal insufficiency).^7^ Hyperpigmentation of the skin can arise in primary adrenal insufficiency, it was not present in our case due to low levels of ACTH. Given the deficiency is in the pituitary and not adrenal gland, there is still mineralocorticoid production. As such hyperkalaemia is usually not present in these patients as was also seen in our case. However despite adequate mineralocorticoid production, it is interesting that patients with ACTH deficiency can still develop hyponatremia due to impairment in vasopressin action and secretion. Hyponatremia can be the initial manifestation of ACTH deficiency as was seen in our case.^8^

Given the variability in definition and no set diagnostic criteria, it is difficult to know the prevalence of isolated ACTH deficiency. Whilst ACTH deficiency can be seen commonly in diffuse pituitary diseases, isolated ACTH deficiency is uncommon,^9^ albeit increasing in number due to the increasing use of immune checkpoint inhibitors.^10^ These immunomodulatory antibodies have substantially improved prognosis for patients with various malignancies such as melanoma but come with risk of serious side effects. Immune‐related adverse events with checkpoint inhibitors are thought to occur through immunologic enhancement and are capable of causing multiple endocrinopathies as side effects.

Case reports have described isolated ACTH deficiency in the setting of traumatic brain injury,^11^ post subarachnoid hemorrhage,^12^ an empty sella,^13^ and in lymphocytic hypophysitis,^14^ however we believe this is the first case report to find isolated ACTH deficiency in the setting of a RC. The mechanism of RC causing hypopituitarism may be due to fluid extravasation from the cyst causing compression of hormone producing cells in the pituitary gland.^15^ However our case is unusual in that only corticotropic cells were affected whereas usually these are affected late in pituitary disease process with somatotropin cells being one of the first.^16^ This could be secondary to an autoimmune etiology as suggested by various case reports. In patients with isolated ACTH deficiency there evidence of concurrent lymphocytic hypophysitis^14^ and thyroiditis^17^ suggesting an underlying autoimmune process. Further evidence comes from a case report where pituitary antibodies were reportedly seen in almost 50 % of patients with isolated ACTH deficiency.^18^ Sauter et al. studied the pathogenesis of isolated ACTH deficiency and suggested that lymphocytic infiltration was usually present.^19^

Hypophysitis tends to show an increase in pituitary gland size with thickening of the pituitary stalk upon brain imaging.^20^ These findings were not seen in our case. Immune check point inhibitors can cause isolated ACTH deficiency without pituitary enlargement in addition to thyroiditis.^21^ It could be the case that RC induced ACTH deficiency follows a similar pathophysiology.

In conclusion we present an unusual and rare case of a RC causing isolated ACTH deficiency. Whilst RC has been seen to cause panhypopituitarism previously, to our knowledge this is the first report to show an isolated ACTH deficiency due to a RC. Our patient was treated with glucocorticoid replacement which resulted in symptomatic and biochemical improvement.

## 7 PATIENT'S PERSPECTIVE

“After returning from a 3‐month stay in England in October 2015, I began to notice losing weight. Approximately 3 years later I visited a General Practitioner for a severe upper respiratory infection which was successfully treated with antibiotics. On my follow‐up appointment I was weighed and subsequently found to have 15 kg weight loss compared to my weight in 2015.

I was referred for an MRI of the pituitary gland which I completed; however, I was lost to follow‐up. In 2018, after seeing another general practitioner, I was referred to an endocrinologist who organized a repeat pituitary MRI examination. After seeing the results I was since started on nutritional supplementation as well as low dose steroid therapy. After commencing on these treatments I can say I feel much better and that the treatment has been very successful.”

## AUTHOR CONTRIBUTIONS

**Hamza azam:** Conceptualization; investigation; methodology; writing – original draft; writing – review and editing. **Bernard Tuch:** Conceptualization; investigation; methodology; supervision; writing – original draft; writing – review and editing.

## CONSENT

Written informed consent was obtained from the patient to publish this report in accordance with the journal's patient consent policy

## Data Availability

The authors confirm that the data supporting the findings of this study are available within the article.
